# On and off the rocks: persistence and ecological diversification in a tropical Australian lizard radiation

**DOI:** 10.1186/s12862-019-1408-1

**Published:** 2019-03-20

**Authors:** Paul M. Oliver, Lauren G. Ashman, Sarah Bank, Rebecca J. Laver, Renae C. Pratt, Leonardo G. Tedeschi, Craig C. Moritz

**Affiliations:** 1 Environmental Futures Research Institute, Griffith University, 170 Kessels Rd, Nathan, Queensland 4111 Australia; 2 Biodiversity and Geosciences Program, Queensland Museum, South Brisbane, Queensland 4101 Australia; 3 Division of Ecology and Evolution, Research School of Biology, and Centre for Biodiversity Analysis, The Australian National University, 46 Sullivans Creek Road, Acton, ACT 2601 Australia; 4 Johann-Friedrich-Blumenbach Institute for Zoology and Anthropology, University of Göttingen, Göttingen, Germany

## Abstract

**Background:**

Congruent patterns in the distribution of biodiversity between regions or habitats suggest that key factors such as climatic and topographic variation may predictably shape evolutionary processes. In a number of tropical and arid biomes, genetic analyses are revealing deeper and more localised lineage diversity in rocky ranges than surrounding habitats. Two potential drivers of localised endemism in rocky areas are refugial persistence through climatic change, or ecological diversification and specialisation. Here we examine how patterns of lineage and phenotypic diversity differ across two broad habitat types (rocky ranges and open woodlands) in a small radiation of gecko lizards in the genus *Gehyra* (the *australis* group) from the Australian Monsoonal Tropics biome.

**Results:**

Using a suite of approaches for delineating evolutionarily independent lineages, we find between 26 and 41 putative evolutionary units in the *australis* group (versus eight species currently recognised). Rocky ranges are home to a greater number of lineages that are also relatively more restricted in distribution, while lineages in open woodland habitats are fewer, more widely distributed, and, in one case, show evidence of range expansion. We infer at least two shifts out of rocky ranges and into surrounding woodlands. Phenotypic divergence between rocky ranges specialist and more generalist taxa is detected, but no convergent evolutionary regimes linked to ecology are inferred.

**Conclusions:**

In climatically unstable biomes such as savannahs, rocky ranges have functioned as zones of persistence, generators of diversity and a source of colonists for surrounding areas. Phenotypic divergence can also be linked to the use of differing habitat types, however, the extent to which ecological specialisation is a primary driver or secondary outcome of localised diversification remains uncertain.

**Electronic supplementary material:**

The online version of this article (10.1186/s12862-019-1408-1) contains supplementary material, which is available to authorized users.

## Background

Contrasting patterns of biodiversity across spatial and climatic gradients spanning broad regions are a key focus of ecological and evolutionary research [1, 2]. Striking variation in the constitution, relative distribution, and evolutionary distinctiveness of biodiversity can also exist at more local scales, for instance, along elevational gradients [3–5] or between islands of varying size [6, 7]. Where consistent patterns of differences in biodiversity occur across habitats, regions and/or taxa, it raises the possibility that key geological, climatic or ecological factors and processes may be predictably shaping these biodiversity patterns, and may also be identifiable.

An emerging distributional pattern in several parts of the world, especially in tropical areas, is that geologically stable and exposed rocky-range systems such as escarpments, limestones or granite outcroppings are associated with localised phylogenetically divergent and/or specialised lineages [8–10]. In contrast, surrounding and often more continuous habitats such as woodlands or forests tend to have much more widespread lineages [11–14]. Two, not necessarily exclusive, processes may explain contrasting patterns of diversity and distribution between rocky ranges and surrounding habitats: 1) refugial persistence in microrefugia such as deep crevices, caves and/or gorges in rocky areas that are buffered from the extremes of climate [15, 16], and 2) divergent selection and localised adaptation leading to the evolution of ecological specialists that use different habitats [17, 18]. While these two processes may function synergistically, they may also generate some contrasting predictions. The refugial hypothesis would predict that the timings of isolation of saxicoline (rock-dwelling) taxa should correspond with periods of climatic stress such as aridification or increasing seasonality. These isolated taxa/lineages may also be predicted to have disjunct distributions, with relatives occurring in other putative rocky refugia, and/or other areas with more mesic climates [15, 19]. If ecological diversification and habitat specialisation is driving localised diversity, phenotypic adaptations linked to occupying different niches may be predicted [20], and ecologically divergent sister species should occur in the neighbouring habitats [21].

Here we examine temporal and spatial patterns of lineage and phenotypic diversity across two broad habitat types in a single lizard radiation in the Australian Monsoonal Tropics (AMT). The AMT spans northern Australia from the Kimberley region in the west, to Cape York in the east. The biome is characterised by a tropical savannah climate with a short, hot and high-rainfall wet season over the austral summer, and a longer warm to hot dry season with little to no rainfall [22]. The AMT landscape has only low (mostly < 500 m) ranges and plateaus and is geologically stable. However, recent palaeoecological analyses point to a highly dynamic climate history that mirrors that of the arid zone to the south – shifting from relatively arid (end Miocene) to mesic (early to mid-Pliocene), then back towards aridity with an increasingly marked monsoon (Late Pliocene and Pleistocene) [23, 24]. During the last glacial maximum, evidence also points to a weakened monsoon, increased aridity and potential contraction of woodlands to the north, to be replaced by grasslands or steppe [25, 26].

Within the AMT savannah biome there are two broad habitat types. The majority of the region consists of relatively flat open woodlands with varying densities of trees. Nested within these woodlands are numerous low (generally at most a few hundred metres above the surrounding landscape) and, to varying degrees, isolated rocky ranges and plateaus of varying size [22]. While these ranges are generally not high enough to generate their own climate, many AMT taxa nonetheless show evidence of localised and often long-term persistence in rocky ranges, especially in areas of sandstone or limestone karst [9, 27–29]. Comparatively, taxa in surrounding savannahs and open woodlands have wider distributions, often shallower genetic divergences and sometimes show evidence of expansion [29, 30]. Broadly, there is also an emerging pattern that lineages in higher rainfall zones of the northern AMT have smaller distributions than relatives in the more arid deserts to the south [27, 31]. Patterns of morphological variation in lineages that occur across rocky ranges and surrounding woodlands in the AMT are less well known. However, in one radiation convergent evolution of specialised rock- and tree-dwelling ecomorphs has been inferred [32], providing evidence that adaptation and ecological diversification may also have played a role in shaping differing patterns of morphological diversity across habitats.

**Fig. 1:**
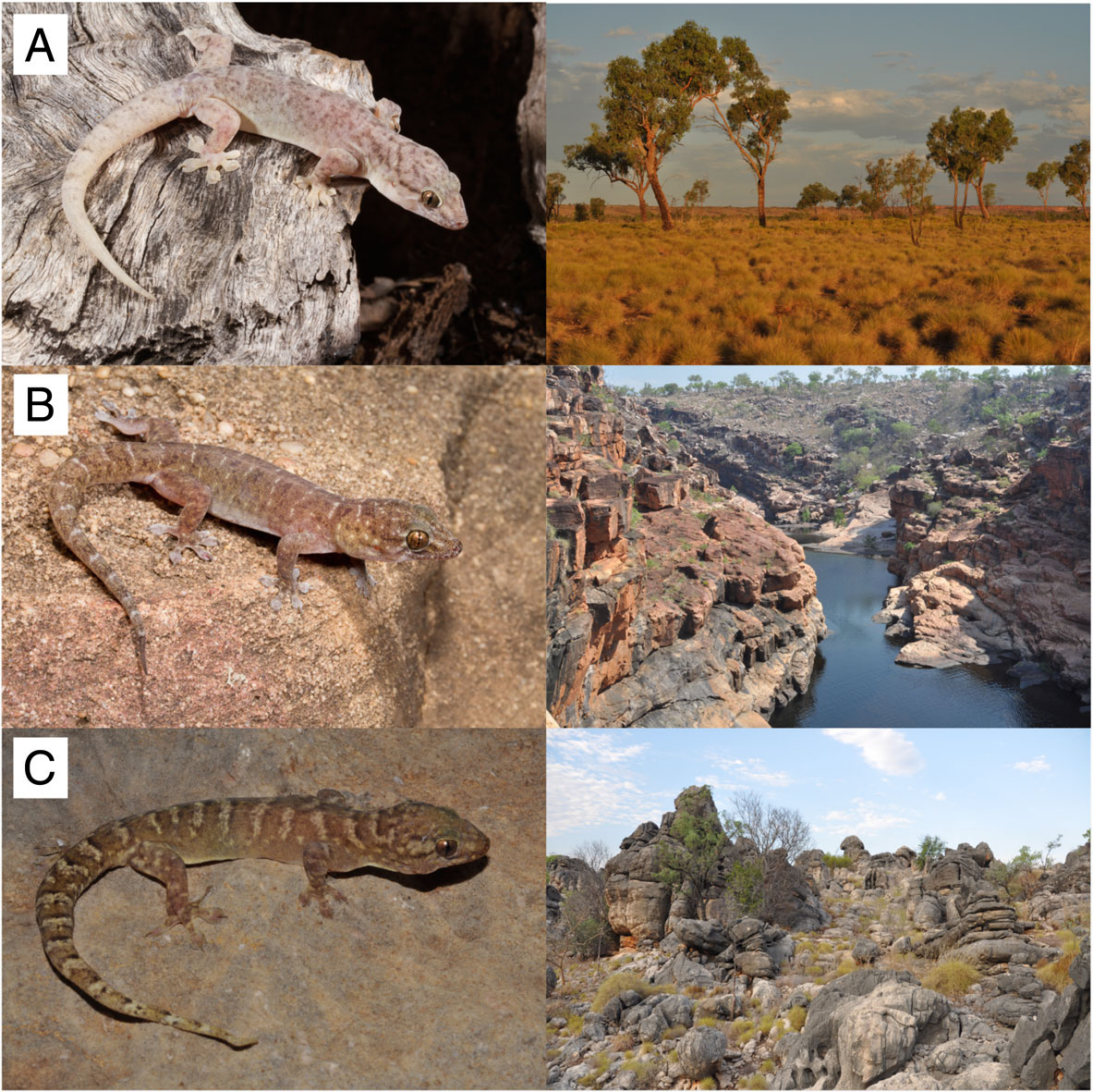
Representative geckos in the *australis* group and their habitats. **a**) *Gehyra australis* from open woodlands, **b**) *Gehyra robusta* from sandstone ranges, and **c**) *Gehyra koira* from limestone ranges. Photographs by Paul Oliver, Paul Horner and Stephen Zozaya

The scansorial geckos in the *australis* group (genus *Gehyra*), currently comprise eight recognised species. Five species are only found in close association with rocky ranges and escarpments (Fig. 1; Additional file 1) and use rock crevices as daytime retreats, even though some forage on trees as well as rocks at night; we refer to these taxa as ‘saxicoline’ [30]. Three species in this complex are most often found on trees in open country with no rocky microhabitats (Fig. 1; Additional file 2), but can forage on rock surfaces in the absence of saxicoline taxa. These are referred to here as habitat ‘generalists’ following recent convention [14, 34]. Other smaller-bodied species of *Gehyra* co-occur in the AMT as part of the phylogenetically distant *variegata* group, but these are almost entirely restricted to rocky habitats and show deep phylogeographic structure [35]. Similarly, the distinct Australian Arid Zone (AAZ) radiation of the *variegata* group has deep phylogeographic diversity in the Pilbara craton (central Western Australia [WA]) and Central Uplands (central Australia) refugia, contrasting with widespread generalist taxa in the rest of the AAZ [14, 36]. An initial multilocus phylogeographic analysis of *australis* group taxa in the eastern half of the AMT (Northern Territory [NT]–Queensland [QLD]) revealed substantial phylogeographic structure in both saxicoline and generalist taxa in the rocky ranges of the Carpentarian sandstones and Selwyn Range, providing evidence that this region has functioned as an evolutionary refugium at the AMT/AAZ interface [37].

Here we build on this previous work by a) expanding sampling to include all taxa in the *australis* group from across the AMT, b) strengthening the phylogenetic framework using an exon capture approach, and c) incorporating phenotypic data into analyses of ecological diversification. Using this dataset, we first test the hypothesis that within a single radiation, rocky ranges would support more localised genetic and lineage diversity than surrounding woodlands. We then test for refugial dynamics as a potential driver of endemism within rocky ranges by estimating the pattern and timing of isolation in rocky habitats, and how this corresponds with late Quaternary climatic change. Finally, to investigate the extent to which adaptive ecological diversification has occurred we tested for phenotypic divergence between habitat types (saxicoline vs. generalist) and convergence within habitat types, trends that have both been detected in a co-distributed lizard lineage [32].

## Methods

### mtDNA assessments of diversity

**Fig. 2:**
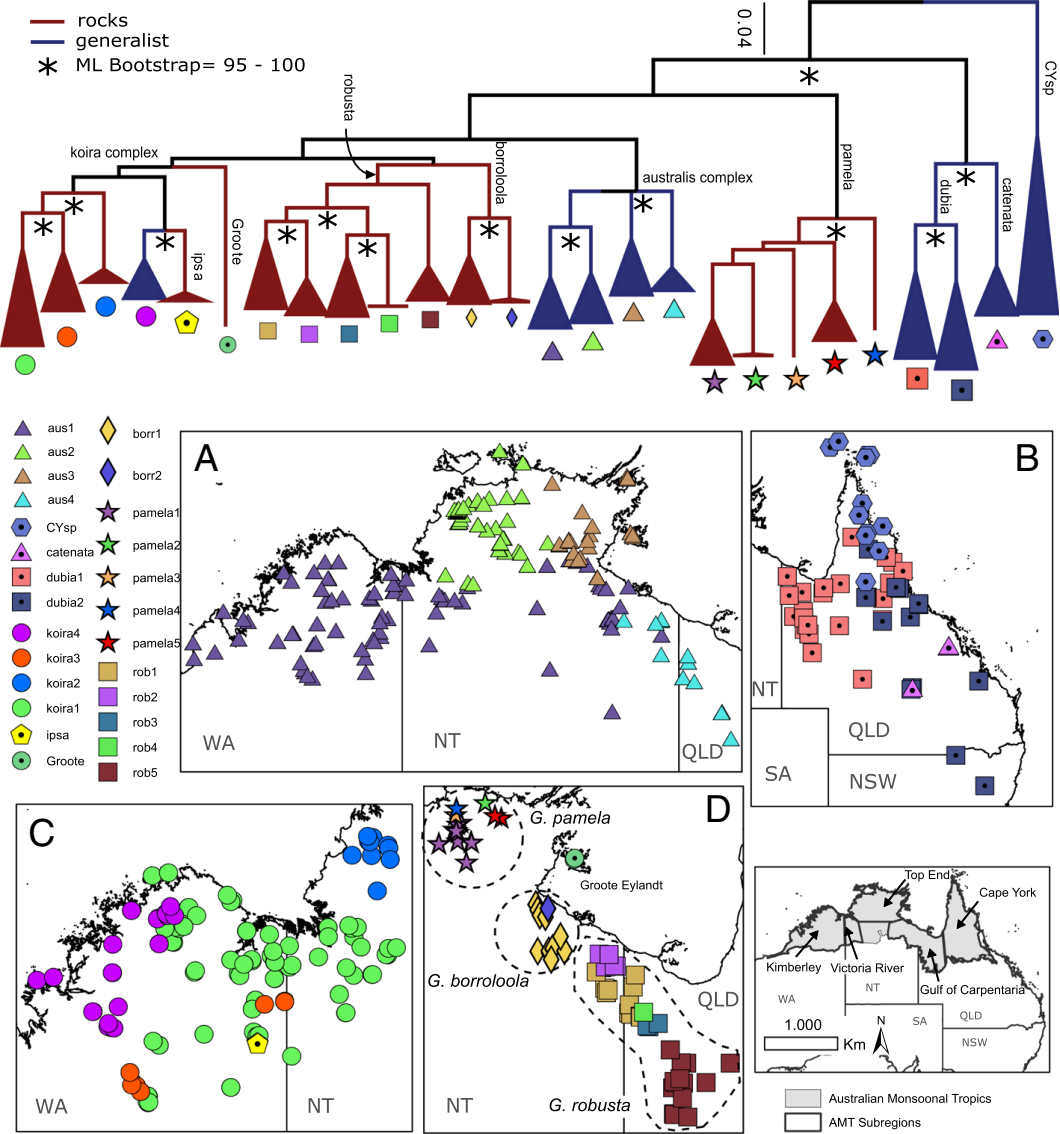
Top: Summary maximum likelihood tree (with bootstrap supports) of mitochondrial (mtDNA) lineage diversity from 774 samples in the *Gehyra australis* group, with lineages colour-coded by ecology (red = saxicoline [rocks only], blue = generalist). Bottom: Distribution and sampling of major mtDNA lineages in the *australis* group across the Australian Monsoonal Tropics (AMT): **a**) *G. australis* complex [AMT], **b**) CYsp/*G. catenata/G. dubia* clades [QLD], **c**) *G. koira* complex [Kimberley/Victoria River regions], and **d**) *G. borroloola-G. robusta*/*G. pamela*/Groote clades [Top End/Gulf region]. Scale bar of phylogenetic tree corresponds to substitutions per site. WA = Western Australia, NT = Northern Territory, QLD = Queensland, NSW = New South Wales, SA = South Australia

For an initial assessment of evolutionary diversity and relationships we amplified the full mitochondrial (mtDNA) *ND2* gene using primers and protocols outlined in detail elsewhere [38]. Our sampling for the *australis* group included 774 unique individuals (Additional file 3: Appendix 1), comprising the majority of pre-existing samples from museum collections across Australia, and an extensive collection of new material from recent fieldwork by ourselves and collaborators across the AMT. We primarily focused on the more densely sampled Gulf of Carpentaria [NT–QLD], the Top End [NT], Victoria River [NT] and the Kimberley [WA] regions, but also included samples from related taxa in more poorly sampled areas of southern New Guinea, the Torres Strait Islands, and eastern Queensland (Fig. 2). Sequence information for newly sampled specimens is lodged on GenBank (accessions MK612781 – MK613064).

Within the mtDNA dataset, we first identified candidate lineages using the shallowest net divergence between two recognised taxa (*Gehyra ipsa* and *G. koira*) as a cut off. To provide a more objective test, we also quantified phylogeographic structure using bGMYC v1.0.2 [39]. We ran bGMYC for 100 MCC chains on a maximum likelihood tree estimated from all unique haplotypes using RAxML v8.2.8 [40] and made ultrametric using the R package phangorn v2.4.7 [41]. bGMYC provides a posterior probability that two sequences belong to the same interbreeding population, which, along with a probability threshold, can be used to determine the number of clusters present. We only recognised clusters above a relatively high posterior probability threshold of 0.9. Using lineages identified by these methods as a guide, we then selected multiple geographically dispersed individuals (mean *n* = 3) from each candidate lineage for genomic analysis via exon capture. Exon capture sampling was particularly focused on divergent mtDNA lineages from areas of parapatry where hybridisation and other forms of introgression would be expected if lineage divergence (speciation) were incomplete.

### Exon capture

Methods for generating and processing exon capture data, and for downstream estimation of phylogenetic trees largely follow those outlined elsewhere [35]. Exon capture probes targeted four commonly used phylogenetic genes *BDNF*, *C-mos*, *PDC* and *RAG1*, and 1716 other protein-coding exon regions identified from *Gehyra nana* [42] and *G. oceanica* [43] transcriptomes. Probes were designed against target exons and synthesised as a SeqCap EZ NimbleGen in-solution capture system. Previous studies have demonstrated that such probes are highly effective for target enrichment across clades considerably older than *Gehyra* [44]. The pooled sample library was hybridised to these probes and amplified by ligation-mediated PCR using the SeqCap EZ Developer Library (NimbleGen) protocol, modified to include the alternative blocking oligonucleotides [45]. A quantitative PCR was run on aliquots of both pre- and post-hybridisation libraries, to ensure the hybridisation reaction had amplified the two target primers but not the non-target control. The successful post-hybridisation library was sequenced using an Illumina HiSeq2000 system at the Biomolecular Resource Facility, John Curtin School of Medical Research, ANU.

### Exon selection, alignment and concatenated tree estimation

Raw sequencing reads were cleaned and trimmed using the *pre-cleanup* and *scrubReads* scripts of the SCPP pipeline [45], using *E. coli* (K12 MG1655 [46]) and human (GRCh37; Ensembl release 67) genome sequences as contaminant references. Regions flanking the target exons were excluded, cleaned reads were assembled into contigs using a pipeline described in [44], and filtering to base quality >20x and sequence coverage >20x, individually assembled, phased, aligned, and concatenated as described elsewhere [35]. Ambiguously aligned sequence sections and codon columns mainly composed of gap symbols were removed [35].

We obtained exon capture data for 1–11 specimens for most major lineages identified by mtDNA (the exceptions were two mtDNA lineages of *G. pamela* for which we did not have samples of sufficient quality for exon capture). The final exon capture dataset included 69 samples from the *australis* group, plus eight outgroup samples (for a total of 77 samples in the overall alignment; see Additional file 3). To gain initial insight into phylogenetic relationships, we used IQ-TREE v1.4.4 [47] to perform a maximum likelihood tree search on the 1634 gene supermatrix; 86 loci were excluded because they had insufficient variation to evaluate substitution models. We then searched for the best-fit substitution model using the option “–m TESTONLY” and applied the HKY substitution model (with 100 bootstrap replicates and random starting trees).

### Exon capture lineage delineation

To identify evolutionarily divergent lineages in the exon capture dataset, we used tr2 [48] for discovery and BP&P v3.4 [49] for validation. tr2 assesses observed vs. expected rates of gene tree congruence under coalescent theory for rooted triplets of candidate taxa and a specified guide tree. For this, we used IQ-TREE gene trees generated for the coalescent-based method ASTRAL-II (556 exons with > 90% complete data; see species tree estimation below) and rooted them with the distantly related *Gehyra mutilata* [50]. Seven gene trees did not contain *G. mutilata* and were therefore discarded. The 549 rooted gene trees together with an IQ-TREE concatenated phylogeny (see above) as a guide tree were then used by tr2 for lineage discovery.

For the more computationally intensive BP&P validation method [49] we selected 50 loci that contained all individuals and were long (≥693 bp, as a proxy for information content) from the refined alignments and included one haplotype from each of two specimens per lineage (best coverage, *n* = 48). We focused on testing support for major groupings that were identified across all datasets. Priors mostly followed [51] (small ancestral population and shallow divergence times), however, because newer versions of BP&P use inverse gamma as priors (instead of gamma priors), we set theta: G(3, 0.002) and tau: G(3, 0.002). As recommended for any coalescent analysis, we ran two independent analyses, but with different starting seeds. To save computational time, we ran independent analyses for each clearly monophyletic species complex (*G. australis*, *G. borroloola*/*robusta*, *G. koira* and *G. pamela* complexes). Fine-tuning parameters were used as the default and analyses were run for a total of 5,000,000 generations, with burn-in set to 10,000 and sampling every five iterations.

### Species tree estimation

Coalescent methods of phylogeny estimation have advantages over concatenation approaches, especially in young radiations [52, 53]. To take advantage of the large number of loci for which we had data, we first used the multi-individual version of ASTRAL-II v4.8.0 [54] to perform tree estimation and multilocus bootstrapping (option ‘–r’) under the multi-species coalescent model using sets of gene and bootstrap trees as input. In order to attain a more complete and informative dataset, loci with less than 90% of all samples and shorter than 300 base pairs were removed. After removal of alignments that did not meet these criteria, 569 loci remained. These were used to create gene trees and bootstrap trees (100 replicates) in IQ-TREE, selecting the HKY substitution model suggested by the IQ-TREE standard model selection (−m TESTONLY option). Due to the high number of invariant sites in some alignments, 13 loci could not be processed successfully, leaving an ASTRAL input of 556 IQ-TREE gene and bootstrap trees. We also quantified the extent of gene tree discordance for the 556 loci dataset by using the ‘-q’ option of ASTRAL-III v5.6.2 [55] to estimate the normalized quartet tree score for the existing tree, as well as the quartet scores for individual nodes.

We also inferred a species tree with StarBEAST2 v0.13.1 [56] implemented in BEAST2 [57]. Given computational constraints, we focused on the same reduced set of 50 loci as the BP&P analyses (see above). The StarBEAST2 analysis was set up in BEASTmasteR v0.1 [58] and run for 100,000,000 generations under the HKY model, calibrated Yule model, and strict lognormal clock. To calibrate the StarBEAST2 analyses we used a secondary age prior (mean 8.5 Mya, SD 2.0) on the node spanning the *G. australis* to *G. dubia* complexes taken from a fossil-calibrated analysis of a five gene dataset spanning geckos (alignment from [59], analysis from [9]). Dating analyses of other nuclear gene datasets that used different fossil calibrations, e.g. [60], have estimated similar ages for this same node in *Gehyra*. Nonetheless, given a suite of potential issues associated with estimating lineage divergence ages from phylogenetic data (e.g., rate variation, tip-age inflation and accuracy of fossil placement and aging), all age estimates should still be treated with caution.

### Range size, genetic diversity and demographics across ecology

Species’ habitats were categorised as saxicoline (shortened to “rock”) or generalist (“gen”) following previous convention [14, 34]. This coding captures broad distributional patterns, rather than absolute microhabitat usage rules. Saxicoline taxa use rock crevices as daytime retreats but can forage on trees as well as rocks and are never found away from rocky ranges. Generalists are most typically observed on trees (or buildings) and all range widely into open woodland areas well removed from rocky ranges, but can also be observed foraging on rocks, especially when specialised saxicoline taxa are absent.

Range sizes for each candidate taxon were estimated using convex hulls estimated in ArcGIS v10 [61] around genetically (mtDNA) typed samples, and where relevant (i.e., for limestone-restricted taxa) further constrained by the extent of suitable habitat. For lineages known from very small areas (*G. ipsa*, plus nine newly identified lineages) we used a 5 km radius buffer around known occurrence sites.

Using the mtDNA dataset we estimated average Tamura-Nei corrected pairwise sequence divergence between and within lineages using MEGA v7.0.26 [62]. DnaSP v5.10.01 [63] was used to calculate various test statistics for population expansion – Tajima’s *D* [64], Fu’s *Fs* [65], and Ramos-Onsins and Roza’s R2 value [66], with *P*-values estimated from coalescent simulations of a 95% confidence interval. For nuclear data, sample sizes per lineage and locus were too small to permit robust tests for population expansion.

Following [67], we calculated the diversification rate (DR) statistic for each taxon (tip) using the StarBEAST2 tree. DR is a summary statistic of the speciation rate derived from the inverse of the branch lengths (i.e., number of splitting events) leading to the particular tip on the tree. We used Phylogenetic Generalized Least Squares (PGLS) in the R package CAPER v0.5.2 [68] and student’s T-tests to test for differences in the log-transformed DR statistic between habitat types, both within the focal radiation from the AMT (i.e., excluding poorly-sampled eastern taxa), and across the entire *australis* group.

### Ancestral states analysis for habitat use

The evolutionary trajectory of habitat use was estimated using BioGeoBEARS v0.2.1 [69]. Six standard biogeographical models were run on the habitat dataset (i.e., saxicoline vs. generalist) in BioGeoBEARS: DEC [70], DIVALIKE [71], and BAYAREALIKE [72], and each with a “+*j*” variant. In addition, a Markov-k model [73] was also run; the model was constructed in BioGeoBEARS by editing the default DEC model by fixing the parameters *d*, e, and *j* to 0, setting the parameter *a* (for anagenetic range-switching) to be free, and eliminating from the state space the null range and any ranges comprising more than one area. All models were run on the species tree for the *australis* group estimated from StarBEAST2 as this provides more accurate estimation of branch lengths, especially towards the tips [52]. The outgroup to the *australis* group is most likely the ecologically diverse and widespread *variegata* group (Moritz et al. unpublished), and even this remains unconfirmed, so we restricted these analyses to the *australis* group alone. Models were fit using maximum likelihood, and the fit was compared with AICc (Akaike’s information criterion corrected for sample size) model weights [74]. Ancestral state estimates were made under each model.

### Morphological data and analyses

Mensural and meristic data were taken from a total of 231 specimens, 87% of which were typed for mtDNA. Some specimens from taxonomically unambiguous localities were included to increase sample sizes for four taxa: *G. catenata*, *G. dubia*, *G. ipsa* and *G. pamela* (Additional file 3). We attempted to include all lineages that were recovered with high support in the exon-based phylogenetic analyses, however, there were insufficient specimens for four lineages (Groote, pamela 4 and 5, robusta 4), and in two instances pairs of ecologically similar sister lineages were pooled to increase sample sizes (borroloola 1 + 2 and dubia 1 + 2). Twelve ecologically relevant morphological traits were measured, following [75]: snout-vent length (SVL), trunk length (TrunkL; body length between legs), trunk width (TrunkW; width between ventral skin folds of forelegs), foreleg length (ForelegL; elbow to heel of palm), hindleg length (HindlegL; knee to heel of palm), head length (HeadL; tip of snout to anterior edge of ear), head depth (HeadD; at deepest point behind eyes), head width (HeadW; at widest point behind eyes), snout length (SnoutL; anterior edge of eye to tip of snout), snout depth (SnoutD; deepest part of head in front of eyes), toe length (ToeL; base of toe to tip of toe pad), and number of lamellae on fourth right toe pad (excluding distal wedge). Tail dimensions were not recorded as tails were often damaged or missing in museum specimens. Measurements were made to the nearest 0.01 mm using digital callipers (right-hand side of the body, dorsal view).

All statistical analyses were performed using the log-transformed intra-lineage means for each trait using the R packages Picante v1.6–2, Phytools v0.5–38, GEIGER v2.0.3 and NLME v3.1–122 [76–79]. Traits other than SVL were corrected for size by regression against SVL (with the residual values being used in analyses). Previous studies have shown that sexual dimorphism is not pronounced in *Gehyra* [14] so we pooled data from males and females.

To initially explore patterns in the phenotypic data, non-phylogenetic principal component analyses (PCAs) were performed on the size-corrected morphological traits (SVL was excluded). PCAs were visualised using phylo-morphospace plots (Phytools), where the phylogeny is overlaid on the plot of morphological variation. Phylogenetic PCA methods were not used as their effectiveness when models of trait evolution differ has been criticised [80]. One-way analyses of variance (ANOVAs) were used on the major PCAs to compare morphology among lineages, with habitat (saxicoline vs. generalists) as the explanatory factor.

The mean values for each morphological trait were further examined using PGLS with ecology (habitat) as a predictor. These analyses included SVL as this is an important trait in *Gehyra* [35], and size-corrected scores for all other variables. For each trait we tested the fit of either Brownian Motion (BM) or Ornstein-Uhlenbeck (OU) models of trait evolution (comparing AICc scores in GEIGER), and ran PGLS with the model that had the best fit.

Finally, using two comparative approaches implemented in R – SURFACE [81] and l1OU [82] – we evaluated whether lineages that have independently shifted into similar habitats have also converged on similar phenotypic optima. We ran both analyses using species’ means for log-transformed SVL and the size-corrected residuals for each log-transformed trait. SURFACE uses a stepwise corrected AICc approach to fit Hansen models and evaluates the most optimal set of evolutionary regimes and regime shifts. The package l1OU employs LASSO (least absolute shrinkage and selector operator) to determine the optimal number of selective regimes in a phylogeny. l1OU applies different Ornstein–Uhlenbeck models to the phylogeny to determine how many different selection regimes are needed to explain the data, and then tries to collapse regimes together. Convergence is indicated by either identical (collapsed) or very similar sets of OU parameters in distantly related taxa.

## Results

### Lineage delimitation from mtDNA and exon datasets

Within the well-sampled Gulf, Top End and Kimberley regions, we provisionally identified 22 candidate lineages in the mtDNA dataset that showed equal or greater divergence than recognised taxa (Fig. 2). Net sequence divergence among intraspecific lineages varied from 6.6 to 14.0% (mean = 9.3%), in comparison to 6.0% between *G. ipsa* and its sister lineage – koira 4 within *G. koira* (Additional file 4: Table S1)*.* All but one of these divergent lineages fall within species complexes that have previously been described and circumscribed: *G. australis* complex – four lineages, all generalist; *G. borroloola* – two lineages, both saxicoline; *G. koira* complex – five lineages, four saxicoline, one generalist; *G. pamela* complex – five lineages, all saxicoline; and *G. robusta* complex – five lineages, all saxicoline. One additional divergent saxicoline lineage from Groote Eylandt (hereafter “Groote”) off the east coast of the Top End (NT) was not obviously associated with any currently recognised species or species complex (Fig. 2).

In the eastern distribution of the *australis* group our sampling was relatively sparse and we conservatively identified only four generalist lineages: one unrecognised taxon “CYsp” (Cape York and the Trans-Fly region of southern New Guinea), *G. catenata* and *G. dubia* (two lineages).

Lineage delineation using bGMYC on mtDNA identified 41 distinct populations across the entire *australis* group. Populations identified in the *G. australis* (*n* = 4 lineages), *G. borroloola* (*n* = 2) and *G. pamela* (*n* = 5) complexes were identical to those from the ad hoc method outlined above (Additional file 4: Table S2). One additional lineage was delineated in the *G. robusta* complex (*n* = 6). Much finer structuring was identified within the *G. koira* complex, specifically koira 1 (*n* = 7), and the more poorly sampled taxa from eastern Australia – *G. dubia* (*n* = 5) and CYsp (n = 7) complexes.

**Fig. 3:**
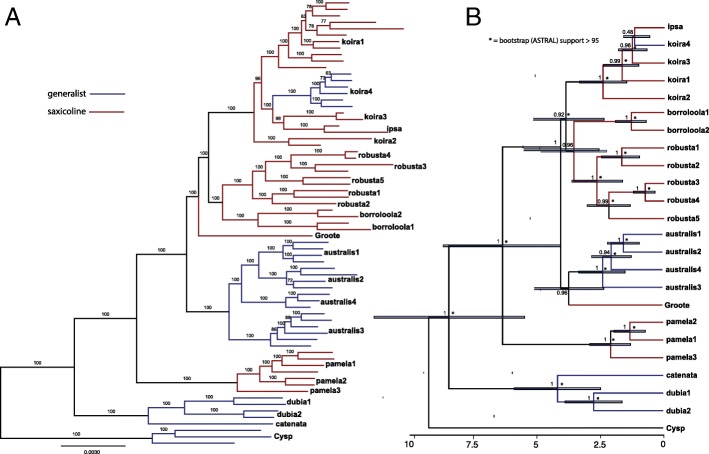
Estimates of phylogenetic relationships within the *australis* group based on exon capture data: **a**) maximum likelihood phylogeny with bootstrap support values estimated using IQ-TREE and 1634 loci for all individuals (scale bar = substitutions per site), and **b**) chronogram with Bayesian support values for major lineages estimated using StarBEAST2 and 50 loci, with nodes also strongly supported by ASTRAL (bootstrap support > 95) demarcated with an asterisk (axis = millions of years before present)

In all trees based on the extensive exon sequences sampled from across the range of the *australis* group (Additional file 5: Figure S1), all recognised species are monophyletic, apart from *G. koira* which is paraphyletic with respect to *G*. *ipsa* (concatenated and species trees; Fig. 3). Furthermore, samples from each of the major mtDNA lineages described above were also monophyletic in the concatenated exon capture phylogeny (Fig. 2a). Species delimitation tests using tr2 on 549 gene trees identified 36 distinct lineages in the *australis* group (Additional file 4: Table S2). In two complexes lineages were entirely congruent with the major mtDNA lineages: *G. borroloola* complex, *n* = 2 lineages; *G. robusta* complex, n = 5 lineages. Additional lineages were identified by tr2 in two other species complexes: *G. australis* complex (n = 5 vs. 4), and *G. pamela* complex (n = 5 vs. 3; noting that two mtDNA lineages were missing from the exon capture dataset for this species). However, in the *G. koira* complex the number of tr2 exon capture lineages greatly exceeded the number of major mtDNA lineages: *n* = 14 vs. 5, including *n* = 7 in koira 1, and *n* = 4 in koira 4. BP&P species delimitation using the exon data supported the distinctiveness of all 22 major candidate lineages identified in the mtDNA dataset with high support (= 1.000). There is also congruence between the mtDNA and the nuclear genome in that no lineages supported by the tr2 analysis of the 549 exons rendered major mtDNA groupings paraphyletic.

In the better sampled Gulf, Top End and Kimberley regions all methods of lineage delimitation identified more evolutionarily distinctive lineages in rocky ranges than surrounding woodlands: 17 saxicoline lineages vs. 5 generalist lineages detected by the ad hoc mtDNA lineages method; 23 vs. 8 using bGMYC of mtDNA; and 22 vs. 9 from exons and tr2. Lineages utilising the same habitat type almost never occurred in sympatry, with the possible exception of the distantly related australis 1 and koira 4 in the Kimberley (Fig. 2). Conversely, saxicoline taxa overlapped with generalist taxa extensively.

### Exon capture phylogeny

Topology and nodes with high support were consistent across all analyses of the nuclear datasets (IQ-TREE, ASTRAL-II, StarBEAST2; Fig. 3; Additional file 5: Figure S2). Two lineages from Queensland—CYsp and *G. catenata*/*G. dubia*—were always recovered as, respectively, the first and second diverging lineage from the remainder of the *australis* group. Remaining taxa from across the Gulf, Top End and Kimberley regions formed a strongly supported clade, within which *G. pamela* from the Arnhem Escarpment (NT) was universally recovered as the sister to a clade comprising the remaining four groups (*G. australis* complex, *G. borroloola* + *G. robusta* complexes, *G. koira* complex, and Groote). Relationships amongst these four groups tended to be more weakly supported and/or inconsistent across methods (Fig. 3; Additional file 5: Figure S2). Patterns of relationships within species complexes were generally consistent and highly supported (Fig. 3). The one exception was three lineages from the southern Kimberley in the *G. koira* complex that formed a polytomy (koira 3, koira 4 and *G. ipsa*), which further contrasted against strong mtDNA support for a close relationship between koira 4 and *G. ipsa*.

The normalized quartet tree score (a potential measure of incomplete lineage sorting [ILS]) for the whole tree calculated in ASTRAL-III was 0.64. For this measure scores closer to 1.00 suggest low levels of ILS. Quartet scores (i.e. percentage of quartets that agree with branch) for nodes varied but were generally high for deeper nodes in the overall *australis* group (> 60%), and lower for nodes at the base of the clade including the *G. australis* complex, *G. borroloola* + *G. robusta* complexes, *G. koira* complex and Groote (Additional file 5: Figure S3).

Based on the secondary age priors we used, three major lineages are estimated to have progressively diverged from the remainder of the *australis* group during the late Miocene: CYsp (mean 9.2 Mya, 95% credible interval [CI] 6.0–12.7 Mya); *G. catenata* + *G. dubia* complex (mean 8.5 Mya, 95% CI 5.5–11.4 Mya); and the *G. pamela* complex (mean 6.3 Mya, 95% CI 4.0–8.7 Mya). More recent Plio-Pleistocene divergences are inferred for initial, and seemingly relatively rapid, radiation of the clade containing all other lineages (mean 4.1 Mya, 95% CI 2.6–5.5 Mya).

### Range sizes, habitat transitions, and demographic history

For the major lineages identified from congruence across mtDNA and exons, saxicoline taxa consistently had ranges an order of magnitude smaller than generalists (Additional file 4: Table S3; mean for all major lineages 12,538 vs. 245,646 km^2^; mean after excluding singleton site lineages with arbitrary ranges 30,541 vs. 245,646 km^2^).

**Fig. 4:**
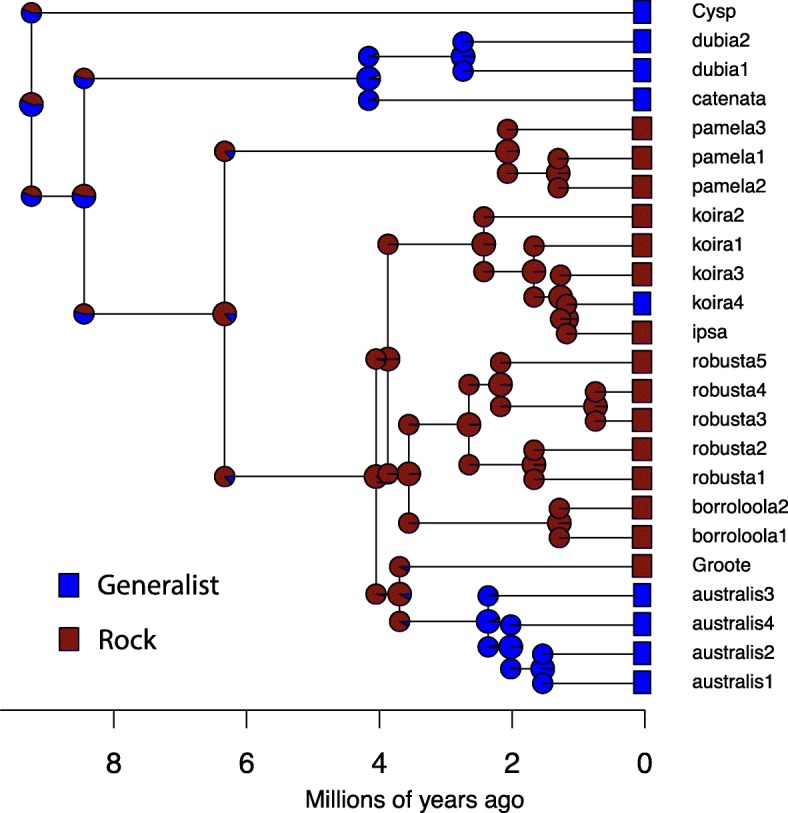
Estimated ancestral states, and number and trajectory of habitat shifts within the *Gehyra australis* group under the Markov-k (MK) model as implemented in BioGeoBEARS (red = saxicoline [rocks only], blue = generalist). Note inferred shifts from saxicoline to generalist ecology at base of *G. australis* complex and within the *G. koira* complex

In analyses of habitat shifts, the Markov-k (MK) model had the best fit (Additional file 4: Table S4). In all analyses multiple shifts between states were inferred (Fig. 4). Habitat preference at the base of the *australis* group was ambiguous, however the saxicoline ecology was supported as basal to the clade containing most species from the Top End to Kimberley regions (Groote plus the *G. australis*, *G. borroloola*, *G. koira*, *G. pamela* and *G. robusta* complexes). Within this radiation at least two more recent and independent shifts from saxicoline to a more generalist habitat use were also inferred: first, from saxicoline lineages to the generalist (and mostly arboreal) *G. australis* complex during the Pliocene; and another more recent (Pleistocene) and more clearly resolved shift from saxicoline to generalist (again mainly arboreal) in a lineage largely endemic to the Kimberley (koira 4; Fig. 4).

Using sequence variation for the better-sampled mtDNA, a genetic signature of population expansion was only found in the *G. australis* complex (generalist), and more specifically, the most widespread lineage within it (australis 1) (*P*-value for Fu’s *Fs* < 0.001; Additional file 4: Table S5).

Across all combinations of taxa (all lineages, eastern lineages excluded) and all statistical tests (PGLS and T-tests) there was no significant difference between saxicoline and generalist habitats in the rate of lineage diversification, as estimated using the DR statistic (Additional file 4: Table S6).

### Morphological variation and evolution

Size-corrected PCA based on residuals (rPCA) suggested differentiation by habitat along PC axis 1 (rPC1; heavily loaded to toe length and head length; Additional file 4: Table S7). ANOVA also detected significant differences between saxicoline and generalist taxa in rPC1 (-HeadL and +ToeL; phyloANOVA *P*-value = 0.015), but not other axes: rPC2 (+HindlegL and -TrunkW) and rPC3 (-SnoutD, -HeadD and -ForelegL; Additional file 4: Tables S7–8).

Univariate analyses of traits using PGLS under either Brownian (BM) or Ornstein-Uhlenbeck (OU) models (whichever was identified as best fit by AICc) found significant differences between the two habitat groups (Additional file 4: Table S9; Additional file 5: Figure S4), with saxicoline taxa tending to be larger (SVL, OU model, *P* = 0.028); with (after size-correction) relatively longer forelimbs (OU, *P* = 0.039); relatively longer heads (BM, *P* = 0.040) and snouts (OU, *P* = 0.008); relatively shallower heads (OU, *P* = 0.031) and snouts (BM, *P* = 0.010); and relatively shorter toes (BM, *P* = 0.008).

**Fig. 5:**
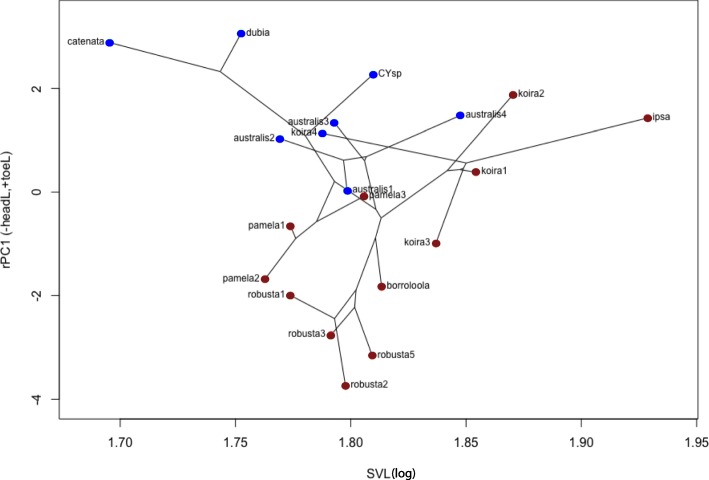
Phylo-morphospace plot of trait means for lineages in the *Gehyra australis* group based on Principal Component Axis 1 (residuals of size-corrected data; rPC1) against log-SVL. Lineages are colour-coded by ecology (red = saxicoline, blue = generalist) and connected by the StarBEAST2 phylogeny

In phylo-morphospace plots of the most variable axes across both univariate and multivariate datasets (logSVL against rPC1; Fig. 5), the *G. koira* complex was the most dispersed, driven by striking divergence in body size (*G. ipsa* large, koira 4 small). Two distantly related saxicoline clades (*G. borroloola* + *G. robusta* complexes, and *G. pamela* complex) overlapped, while saxicoline taxa in the *G. koira* complex were more divergent (much larger and tending to have shorter heads and longer toes). Amongst the generalist taxa the most striking similarity was between koira 4 and relatively distantly related members of the *G. australis* complex (Fig. 5).

SURFACE analyses for convergence based on the entire dataset inferred only two regime shifts, both in saxicoline lineages: a) the relatively long-headed and short-toed *G. borroloola* and *G. robusta* complexes, and b) the very large species *G. ipsa*. No convergent regime shifts were identified (Additional file 5: Figure S5). l1OU analyses identified seven regime shifts, comprising the two above, plus additional candidate lineage-specific shifts in the *G. australis* (*n* = 1), *G. koira* (*n* = 2), *G. pamela* (n = 1) and *G. robusta* (*n* = 1) complexes (Additional file 5: Figure S6). No convergent regimes were identified in either method.

## Discussion

Comprehensive mtDNA sampling of diversity in geckos from the *australis* group (*Gehyra*) combined with a well-resolved tree from an exon capture dataset shows that lineage diversity is higher in rocky ranges than in surrounding woodlands. Saxicoline taxa also occupy geographic ranges that are an order of magnitude smaller than those of generalists, with more than half these saxicoline taxa being short-range endemics (ranges less than 10,000 km^2^) [83]. This result accords with other studies in rainforest, savannah and arid habitats that have identified that localised lizard endemism and diversity is often high in lineages associated with rocky ranges [84–86], with taxa in surrounding habitats often tending to be more genetically homogeneous and/or widespread [8, 13, 14]. Below, we focus on two, not necessarily exclusive, processes that may underpin these contrasting patterns of diversity and distribution across these interdigitated habitat types: 1) refugial persistence in rocky ranges through climatic change, and 2) localised evolution of habitat specialists across striking habitat gradients.

### Refugia – On and off the rocks

Past climatic change has played a critical role in shaping contrasting patterns of distribution and diversity across Australian biomes, regions and habitats [87, 88]. Recent palaeoclimatic data point towards the AMT being a young biome with a dynamic climatic history of arid-mesic-arid cycling that parallels that of the arid zone to the south, shifting from relatively arid at late Miocene, to mesic in the early to mid-Pliocene, then towards increasing aridity and a marked monsoon through the Late Pliocene and Pleistocene [23]. Our estimate of crown age for the radiation of most of the *australis* group across the Gulf, Top End and Kimberley regions is congruent with this mesic pulse in the early Pliocene. Other phylogenetic studies also point to endemic AMT savannah lineages radiating over this timeframe [27, 30, 31, 89], while older relicts, potentially persisting remnants from an earlier (mid-Miocene) wet phase, tend to have highly disjunct distributions and very low diversity [28]. Critically, the majority of lineages of *Gehyra* that we estimate to have diversified through major climatic changes since the Late Miocene are restricted to rocky ranges.

Periods of increasing aridity also seem to have reinforced an overall south-to-north gradient of increasingly old and/or finely structured diversity, even within the rock-associated lineages in both the *australis* group, and other AMT radiations [31, 90]. At one end of the temporal spectrum, to the north a deep (pre-Pliocene) lineage within the main AMT radiation (*G. pamela*) is restricted to the rocky Arnhem Escarpment of the Top End. This topographically complex, relatively high rainfall region is characterised by high phylogenetic endemism in other taxa [8] including isolated lineages that are either absent, or highly relictual across the remainder of the AMT [91, 92]. In the more arid southern AMT, isolated populations of saxicolous lineages from topographically complex ranges in both the southern Kimberley (*G. ipsa*) and the Gulf region (robusta 5), suggest more recent (late Pliocene – early Pleistocene) isolation. The apparently disjunct distribution of koira 3 across the southern and eastern Kimberley limestones implies an even more recent range contraction over relatively large areas, perhaps linked to Pleistocene expansion of desert sand dunes [93] from the south over formerly continuous limestone habitats.

Comparisons of mtDNA diversity in the two widespread taxa in the western AMT provide evidence that ecological differences may have shaped contrasting patterns of persistence. The saxicoline koira 1 has strong internal phylogeographic structure, implying multiple areas of persistence. By contrast, the geographically overlapping generalist (mostly arboreal) australis 1 lineage has effectively no internal structure and shows evidence of recent range expansion (Additional file 4: Table S5). In some other low relief tropical landscapes, rocky ranges appear to have functioned as critical refugia during periods of relative aridity over evolutionary timescales [9, 85, 94]. Localised and higher genetic diversity in rocks also suggests that contemporary variation in endemism across habitats may be underpinned by differing probabilities of extinction in response to climatic change [5]. Accordingly, estimation of species' responses to future climatic change in these habitats will also increasingly need to incorporate the potential role and accessibility of microrefugia [95].

In addition to being refuges, our ancestral state analyses further imply that rocky ranges may be sources of recolonisation into surrounding habitats. In the *G. koira* complex four out of five species are saxicoline, while koira 4, which is deeply nested within the complex, is largely arboreal. Strikingly, koira 4 is similar to co-occurring members of the generalist *G. australis* complex in body size and also shares a plain dorsal pattern, to the extent that fieldworkers consistently cannot distinguish these two taxa. This seemingly recent ecological and phenotypic shift out of rocky ranges occurs in the west Kimberley, a region that marks the eastern distributional limits of many mesic-dependent taxa and is a recognised hotspot of endemism [96]. Estimation of the trajectories of ecological evolution in *Gehyra* elsewhere in Australia [14], and other geckos from Australia [28] and overseas [97] also imply that contraction into rocky refugia may not necessarily be an endpoint. Instead, these habitats are inferred to function as sources of diversity for surrounding habitats when (or where) climates are less challenging. Going forward, a major challenge to testing such a model will be controlling for how non-random or differing rates of extinction and anagenesis across habitat types may bias ancestral state estimation [98].

### Specialisation on the rocks

Ecological differentiation and adaptation to distinctive microhabitats is an alternative process that could generate localised endemism, even in the absence of population isolation and persistence [17]. One prediction of ecological divergence is that taxa in different habitats may show evidence of associated adaptive divergence or specialisation [21]. In overall univariate analyses we found evidence that the saxicoline taxa in the *australis* group were larger than generalists, and had longer and shallower heads, longer forelimbs, and shorter toes. Phylo-morphospace plots also suggested that the distantly related saxicoline *G. borroloola*/*robusta* and *G. pamela* complexes tend to have shorter toes and longer heads. Some of these phenotypic shifts, such as flattened body shape, typify rock-dwelling taxa in other lineages of *Gehyra* [14], other geckos [18], and other radiations of lizards [34, 99, 100]. However, while follow-up SURFACE and l1OU analyses identified divergent morphological regimes, they did not suggest convergence. This contrasts with a geographically overlapping radiation of Australian skinks in the genus *Cryptoblepharus* that shows a very strong link between body shape and ecology, coupled with clear evidence of convergent evolutionary regimes leading to distinct rock and tree ecomorphs [32]. However, recent work does suggest that in *Gehyra*, and at least some other lizard radiations, the link between ecology, phenotype (at least as we have scored these data) and lineage diversity may be complex [101, 102], and not as overt as in some classical adaptive radiations of lizards [103]. In this context, it is safe to argue that some phenotypic diversification linked to ecology has occurred in the *australis* group, but the extent to which ecological diversification may have been a primary driver of higher diversity and localised endemism in rocky ranges in this, and many other systems, remains difficult to test.

### Biotic interactions?

While we focus on refugial dynamics and specialisation above, a third process, biotic interactions, may also play an important role in shaping communities and distributions. For instance, in lizards, specialisations to utilise habitats such as rocks, have been linked to a lowered ability to compete in other habitats via mechanisms such as reduced reproductive output or dietary breadth [34]. Notably, we found that *australis* group taxa with similar habitat preferences almost never overlap geographically, while conversely, saxicoline and generalist taxa are frequently sympatric, even in closely-related lineages such as koira 4 and koira 1. Furthermore, generalist taxa usually use rocks only when saxicolous taxa are absent ([33], Moritz, Oliver, Tedeschi pers. obs.). Recent studies have pointed to rapid shifts in body size and the likely importance of size-based sorting of local assemblages in other lineages of *Gehyra* [35, 75]. In the west Kimberley, the large saxicoline koira 1 lineage also abuts with the distributional range edge of another saxicoline (and large) taxon, *Gehyra xenopus*, while the generalist (and much smaller) koira 4 widely overlaps with *G. xenopus* (authors pers. obs.). These data all support an established pattern that similar-sized and ecologically undifferentiated lizard taxa rarely persist in the same communities [35, 103, 104], underlining the argument that interactions between species play an important role in shaping extant distributional limits [105]. However, testing the extent to which this ecological differentiation is a secondary outcome of speciation events or a primary driver of them remains challenging.

## Conclusions

Across a mosaic of habitats in the savannah of northern Australia, rocky ranges appear to have provided climatically-buffered microrefugia through late Cenozoic aridification, mediating localised persistence, diversification and even recolonisation of surrounding woodlands. This result emphasises the likely importance of microrefugia within rocky habitats for persistence through past – and future – climatic changes. Patterns of phenotypic variation and geographic distributions suggest that ecological specialisation and species interactions may have played a complementary role in shaping contemporary diversity patterns; however, the extent to which these latter processes may be primary drivers of lineage divergence remains ambiguous. Going forward, the challenge remains how to find ways to convincingly disentangle the roles these key processes have, and will, play in shaping diversity patterns through changing climates. Integrative studies linking genetic patterns with a detailed understanding of physiology, habitat preferences and biotic interactions offer the potential to meet this challenge [106].

## Additional files

Additional file 1: Distribution of sampling (in kmz file format) for saxicoline taxa in the *G. australis* group in this paper. (KMZ 6 kb)

Additional file 2: Distribution of sampling (in kmz file format) for generalist taxa in the *G. australis* group in this paper. (KMZ 8 kb)

Additional file 3: **Appendix 1.** Full list of samples included in genetic and morphological analyses inlcuding GenBank accession details. Samples which were assigned to genetic lineages based on locality (no DNA) are indicated with an asterisk. (XLSX 135 kb)

Additional file 4: **Table S1.** Net Tamura-Nei corrected genetic divergences amongst major mtDNA lineages. **Table S2.** Summary species delimitation results using ad hoc mtDNA divergences, bGMYC on mtDNA data, and tr2 on exon capture data. **Table S3.** Estimates of range-size (km^2^) for lineages in the *Gehyra australis* group based on minimum convex hulls for genetically-typed samples. **Table S4.** Relative likelihood and AIC scores for competing models of ecological trait evolution, as implemented in BioGeoBEARS. **Table S5.** Summary of population genetics statistics and expansion tests for major lineages (estimated from mtDNA). **Table S6.** Comparison of mean diversification rate statistic estimates for the *Gehyra australis* group stratified by ecology. **Table S7.** Loadings, variance and percentage of data variance explained from the principal components analyses (PCA) on all log-transformed morphological traits (“sPCA”), and on the residuals of body shape traits against SVL (“rPCA”). **Table S8.** Morphological differentiation between habitat types in the *Gehyra australis* group using multivariate data and ANOVA (phylogenetic or non-phylogenetic, based on tests for phylogenetic signal). **Table S9.** Morphological differentiation between habitat types in the *Gehyra australis* group using univariate data and Phylogenetic Generalized Least Squares (PGLS) regression and most likely model of trait evolution (BM = Brownian Motion, OU = Ornstein-Uhlenbeck). (DOCX 64 kb)

Additional file 5: **Figure S1.** Geographic distribution and lineage allocation for samples from the *Gehyra australis* group included in exon capture. **Figure S2.** Topology and support values estimated by ASTRAL-II from 556 IQ-TREE gene trees for the *Gehyra australis* group. **Figure S3.** ASTRAL-III quartet support mapped onto species tree for the *Gehyra australis* group. **Figure S4.** Mean and ranges of values for morphological characters that showed evidence of significant variation across saxicoline and generalist taxa (according to PGLS) in the *Gehyra australis* group. **Figure S5.** Morphological regime shifts within the *Gehyra australis* group inferred by SURFACE based on an analysis including SVL (log-transformed) and 11 other size-corrected morphological characters. **Figure S6.** Morphological regime shifts within the *Gehyra australis* group inferred by l1OU based on an analysis including SVL (log-transformed) and 11 other size-corrected morphological characters. (DOC 999 kb)

## Abbreviations

AAZ: Australian Arid Zone
AICc: Akaike’s Information Criterion corrected
AMT: Australian Monsoonal Tropics
ANOVA: Analysis of Variance
BM: Brownian Motion
DR: Diversification Rate
mtDNA: mitochondrial DNA
NSW: New South Wales
NT: Northern Territory
OU: Ornstein-Uhlenbeck
PCA: Principal Component Analysis
QLD: Queensland
SA: South Australia
SVL: snout-vent length
WA: Western Australia
