# Supplementary material for: On and off the rocks: persistence and ecological diversification in a tropical Australian lizard radiation
Source: BMC Evol Biol. 2019 Mar 20;19:81. doi: 10.1186/s12862-019-1408-1 (PMC6427882; doi:10.1186/s12862-019-1408-1)
Supplement: Supplementary file 4 — Table S1. Net Tamura-Nei corrected genetic divergences amongst major mtDNA lineages. Table S2. Summary species delimitation results using ad hoc mtDNA divergences, bGMYC on mtDNA data, and tr2 on exon capture data. Table S3. Estimates of range-size (km2) for lineages in the Gehyra australis group based on minimum convex hulls for genetically-typed samples. Table S4. Relative likelihood and AIC scores for competing models of ecological trait evolution, as implemented in BioGeoBEARS. Table S5. Summary of population genetics statistics and expansion tests for major lineages (estimated from mtDNA). Table S6. Comparison of mean diversification rate statistic estimates for the Gehyra australis group stratified by ecology. Table S7. Loadings, variance and percentage of data variance explained from the principal components analyses (PCA) on all log-transformed morphological traits (“sPCA”), and on the residuals of body shape traits against SVL (“rPCA”). Table S8. Morphological differentiation between habitat types in the Gehyra australis group using multivariate data and ANOVA (phylogenetic or non-phylogenetic, based on tests for phylogenetic signal). Table S9. Morphological differentiation between habitat types in the Gehyra australis group using univariate data and Phylogenetic Generalized Least Squares (PGLS) regression and most likely model of trait evolution (BM = Brownian Motion, OU = Ornstein-Uhlenbeck). (DOCX 64 kb) [file 12862_2019_1408_MOESM4_ESM.docx]

*BMC Evolutionary Biology*

**ADDITIONAL FILE 4**

**On and off the rocks: persistence and ecological diversification in a tropical Australian lizard radiation.**

Paul M. Oliver, Lauren G. Ashman, Sarah Bank, Rebecca J. Laver, Renae C. Pratt, Leonardo G. Tedeschi and Craig C. Moritz

**Additional file 4:** mitochondrial divergences between major lineages (Table S1); summary species delimitation using different datasets and methods (Table S2); range-size estimates for major lineages (Table S3); BioGeoBEARS model comparisons for ecological trait evolution (Table S4); mitochondrial diversity and tests of population expansion (Table S5); diversification rate estimates for ecology (habitat type) across the phylogeny (Table S6); principal component loadings for PCAs on morphological trait variation (Table S7); results of multivariate morphometric analyses and ANOVA tests to assess morphological differentiation between habitat types (Table S8); results of univariate Phylogenetic Generalized Least Squares regression tests on morphological traits to assess morphological differentiation between habitat types (Table S9).

**Table S1. Net Tamura-Nei corrected genetic divergences (*ND2*) between major mtDNA lineages within the *Gehyra australis* group**. Minimum genetic divergence between lineages highlighted in red.

|  | **koira1** | **koira2** | **koira3** | **koira4** | ***ipsa*** | **Groote** | **borr1** | **borr2** | | **rob1** | | **rob2** | | **rob3** | **rob4** | | **rob5** | **aus1** | **aus2** | **aus3** | **aus4** | **pam1** | **pam2** | **pam3** | **pam4** | **pam5** | ***catenata*** | **dub1** | **dub2** | |  |  |
| --- | --- | --- | --- | --- | --- | --- | --- | --- | --- | --- | --- | --- | --- | --- | --- | --- | --- | --- | --- | --- | --- | --- | --- | --- | --- | --- | --- | --- | --- | --- | --- | --- |
| **koira1** |  |  |  |  |  |  |  |  | |  | |  | |  |  | |  |  |  |  |  |  |  |  |  |  |  |  |  | |  |  |
| **koira2** | 0.080 |  |  |  |  |  | |  |  | |  | |  |  | |  | |  |  |  |  |  |  |  |  |  |  |  | |  | |  |
| **koira3** | 0.064 | 0.090 |  |  |  |  |  |  | |  | |  | |  |  | |  |  |  |  |  |  |  |  |  |  |  |  |  | |  |  |
| **koira4** | 0.083 | 0.103 | 0.104 |  |  |  |  |  | |  | |  | |  |  | |  |  |  |  |  |  |  |  |  |  |  |  |  | |  |  |
| ***ipsa*** | 0.098 | 0.120 | 0.115 | **0.060** |  |  |  |  | |  | |  | |  |  | |  |  |  |  |  |  |  |  |  |  |  |  |  | |  |  |
| **Groote** | 0.118 | 0.139 | 0.149 | 0.135 | 0.150 |  |  |  | |  | |  | |  |  | |  |  |  |  |  |  |  |  |  |  |  |  |  | |  |  |
| **borr1** | 0.099 | 0.102 | 0.109 | 0.113 | 0.120 | 0.142 |  |  | |  | |  | |  |  | |  |  |  |  |  |  |  |  |  |  |  |  |  | |  |  |
| **borr2** | 0.106 | 0.121 | 0.120 | 0.117 | 0.135 | 0.152 | 0.077 |  | |  | |  | |  |  | |  |  |  |  |  |  |  |  |  |  |  |  |  | |  |  |
| **rob1** | 0.087 | 0.103 | 0.103 | 0.094 | 0.116 | 0.133 | 0.089 | 0.112 | |  | |  | |  |  | |  |  |  |  |  |  |  |  |  |  |  |  |  | |  |  |
| **rob2** | 0.116 | 0.133 | 0.133 | 0.125 | 0.142 | 0.157 | 0.122 | 0.131 | | 0.066 | |  | |  |  | |  |  |  |  |  |  |  |  |  |  |  |  |  | |  |  |
| **rob3** | 0.101 | 0.131 | 0.115 | 0.125 | 0.139 | 0.152 | 0.111 | 0.141 | | 0.071 | | 0.094 | |  |  | |  |  |  |  |  |  |  |  |  |  |  |  |  | |  |  |
| **rob4** | 0.110 | 0.130 | 0.128 | 0.131 | 0.146 | 0.166 | 0.126 | 0.141 | | 0.080 | | 0.108 | | 0.078 |  | |  |  |  |  |  |  |  |  |  |  |  |  |  | |  |  |
| **rob5** | 0.105 | 0.119 | 0.117 | 0.108 | 0.123 | 0.145 | 0.110 | 0.118 | | 0.077 | | 0.104 | | 0.101 | 0.115 | |  |  |  |  |  |  |  |  |  |  |  |  |  | |  |  |
| **aus1** | 0.117 | 0.141 | 0.132 | 0.129 | 0.138 | 0.164 | 0.133 | 0.144 | | 0.119 | | 0.137 | | 0.139 | 0.142 | | 0.130 |  |  |  |  |  |  |  |  |  |  |  |  | |  |  |
| **aus2** | 0.123 | 0.134 | 0.130 | 0.129 | 0.153 | 0.163 | 0.145 | 0.152 | | 0.122 | | 0.144 | | 0.139 | 0.146 | | 0.123 | 0.069 |  |  |  |  |  |  |  |  |  |  |  | |  |  |
| **aus3** | 0.112 | 0.126 | 0.121 | 0.124 | 0.147 | 0.144 | 0.126 | 0.134 | | 0.116 | | 0.130 | | 0.130 | 0.150 | | 0.122 | 0.079 | 0.079 |  |  |  |  |  |  |  |  |  |  | |  |  |
| **aus4** | 0.126 | 0.138 | 0.125 | 0.132 | 0.150 | 0.164 | 0.138 | 0.142 | | 0.117 | | 0.145 | | 0.140 | 0.161 | | 0.121 | 0.098 | 0.085 | 0.089 |  |  |  |  |  |  |  |  |  | |  |  |
| **pam1** | 0.171 | 0.194 | 0.169 | 0.181 | 0.190 | 0.208 | 0.181 | 0.20 | | 0.161 | | 0.183 | | 0.164 | 0.183 | | 0.161 | 0.192 | 0.170 | 0.176 | 0.187 |  |  |  |  |  |  |  |  | |  |  |
| **pam2** | 0.198 | 0.220 | 0.195 | 0.204 | 0.218 | 0.226 | 0.205 | 0.217 | | 0.187 | | 0.204 | | 0.185 | 0.214 | | 0.188 | 0.205 | 0.206 | 0.194 | 0.211 | 0.097 |  |  |  |  |  |  |  | |  |  |
| **pam3** | 0.176 | 0.199 | 0.186 | 0.190 | 0.199 | 0.204 | 0.186 | 0.20 | | 0.165 | | 0.183 | | 0.180 | 0.188 | | 0.176 | 0.188 | 0.182 | 0.192 | 0.198 | 0.101 | 0.129 |  |  |  |  |  |  | |  |  |
| **pam4** | 0.167 | 0.190 | 0.176 | 0.182 | 0.193 | 0.202 | 0.181 | 0.195 | | 0.172 | | 0.197 | | 0.176 | 0.196 | | 0.178 | 0.184 | 0.183 | 0.170 | 0.176 | 0.117 | 0.136 | 0.141 |  |  |  |  |  | |  |  |
| **pam5** | 0.154 | 0.174 | 0.164 | 0.161 | 0.174 | 0.171 | 0.165 | 0.167 | | 0.145 | | 0.167 | | 0.161 | 0.171 | | 0.154 | 0.171 | 0.168 | 0.166 | 0.174 | 0.080 | 0.101 | 0.097 | 0.114 |  |  |  |  | |  |  |
| ***catenata*** | 0.153 | 0.177 | 0.181 | 0.176 | 0.197 | 0.193 | 0.183 | 0.182 | | 0.166 | | 0.191 | | 0.181 | 0.193 | | 0.171 | 0.173 | 0.178 | 0.158 | 0.193 | 0.211 | 0.238 | 0.210 | 0.217 | 0.200 |  |  |  | |  |  |
| **dub1** | 0.153 | 0.189 | 0.188 | 0.177 | 0.191 | 0.192 | 0.179 | 0.190 | | 0.167 | | 0.188 | | 0.175 | 0.186 | | 0.161 | 0.182 | 0.175 | 0.162 | 0.188 | 0.190 | 0.221 | 0.193 | 0.201 | 0.190 | 0.107 |  |  | |  |  |
| **dub2** | 0.161 | 0.191 | 0.186 | 0.174 | 0.196 | 0.192 | 0.186 | 0.190 | | 0.164 | | 0.192 | | 0.180 | 0.201 | | 0.162 | 0.178 | 0.173 | 0.163 | 0.186 | 0.200 | 0.224 | 0.201 | 0.216 | 0.196 | 0.119 | 0.076 |  | |  |  |
| **CYsp** | 0.165 | 0.179 | 0.163 | 0.176 | 0.192 | 0.204 | 0.176 | 0.190 | | 0.160 | | 0.180 | | 0.177 | 0.184 | | 0.180 | 0.181 | 0.174 | 0.174 | 0.180 | 0.194 | 0.222 | 0.203 | 0.195 | 0.181 | 0.172 | 0.180 | 0.183 | |  |  |

**Table S2. Summary species delimitation results for each major *Gehyra* complex within the *australis* group.** Calculated using *ad hoc* mtDNA divergence or bGMYC on mtDNA data, and tr2 on exon capture data. *n* = number of individual samples included in analyses.

|  | **mtDNA** | | | |  | **exon capture** | |
| --- | --- | --- | --- | --- | --- | --- | --- |
| **complex** | **mtDNA ad hoc** |  | **bGMYC** | ***n*** |  | **tr2 lineages** | ***n*** |
| ***australis*** | australis 1 |  | 1 | 115 |  | 1 | 4 |
|  | australis 2 |  | 1 | 55 |  | 1 | 4 |
|  | australis 3 |  | 1 | 28 |  | 1 | 6 |
|  | australis 4 |  | 1 | 13 |  | 2 | 3 |
|  |  |  |  |  |  |  |  |
| ***borroloola*** | borroloola 1 |  | 1 | 26 |  | 1 | 2 |
|  | borroloola 2 |  | 1 | 5 |  | 1 | 2 |
|  |  |  |  |  |  |  |  |
| ***koira*** | koira 1 |  | 7 | 142 |  | 7 | 11 |
| **(and *ipsa*)** | koira 2 |  | 1 | 14 |  | 1 | 2 |
|  | koira 3 |  | 1 | 17 |  | 1 | 2 |
|  | koira 4 |  | 4 | 31 |  | 4 | 6 |
|  | *G. ipsa* |  | 1 | 4 |  | 1 | 2 |
|  |  |  |  |  |  |  |  |
| ***pamela*** | pamela 1 |  | 1 | 19 |  | 3 | 4 |
|  | pamela 2 |  | 1 | 6 |  | 1 | 2 |
|  | pamela 3 |  | 1 | 1 |  | 1 | 1 |
|  | pamela 4 |  | 1 | 4 |  | NA | 0 |
|  | pamela 5 |  | 1 | 2 |  | NA | 0 |
|  |  |  |  |  |  |  |  |
| ***robusta*** | robusta 1 |  | 2 | 22 |  | 1 | 2 |
|  | robusta 2 |  | 1 | 9 |  | 1 | 1 |
|  | robusta 3 |  | 1 | 6 |  | 1 | 2 |
|  | robusta 4 |  | 1 | 3 |  | 1 | 2 |
|  | robusta 5 |  | 1 | 35 |  | 1 | 2 |
|  |  |  |  |  |  |  |  |
| ***catenata*** | *G. catenata* |  | 2 | 3 |  | 1 | 1 |
|  |  |  |  |  |  |  |  |
| ***dubia*** | dubia 1 |  | 1 | 33 |  | 1 | 2 |
|  | dubia 2 |  | 3 | 25 |  | 1 | 2 |
|  |  |  |  |  |  |  |  |
| **CYsp** | NA |  | 6 | 28 |  | 1 | 3 |
| **Groote** | NA |  | 1 | 3 |  | 1 | 1 |
|  |  |  |  |  |  |  |  |
| Total lineages | 26 |  | 44 |  |  | 36 |  |

**Table S3. Estimates of distribution size (km^2^) for lineages in the *Gehyra australis* group.** Measurements based on minimum convex hulls for genetically-typed samples. Saxicoline taxa shaded in grey.

| **Lineage** | **Area (km^2^)** | **Habitat** |
| --- | --- | --- |
| aus1 | 641,440.58 | Generalist |
| dubia2 | 480,291.81 | Generalist |
| dubia1 | 464,719.43 | Generalist |
| *G. catenata* | 316,084.02 | Generalist |
| koira1 | 181,853.42 | Saxicoline |
| aus2 | 103,327.89 | Generalist |
| CYsp | 92,802.35 | Generalist |
| aus3 | 40,750.15 | Generalist |
| koira4 | 36,647.87 | Generalist |
| aus4 | 34,753.22 | Generalist |
| rob5 | 24,937.96 | Saxicoline |
| rob1 | 13,057.23 | Saxicoline |
| borr1 | 9,276.91 | Saxicoline |
| pamela1 | 7,349.05 | Saxicoline |
| koira2 | 6,233.34 | Saxicoline |
| rob2 | 1,402.77 | Saxicoline |
| borr2 | 314.12 | Saxicoline |
| Groote | 314.12 | Saxicoline |
| *G. ipsa* | 314.12 | Saxicoline |
| pamela2 | 314.12 | Saxicoline |
| pamela3 | 314.12 | Saxicoline |
| pamela4 | 314.12 | Saxicoline |
| pamela5 | 314.12 | Saxicoline |
| rob3 | 314.12 | Saxicoline |
| rob4 | 314.12 | Saxicoline |
| koira3 | 222.93 | Saxicoline |

**Table S4. Relative likelihoods and AIC scores for competing models of ecological trait (habitat – saxicoline vs. generalists) evolution, as implemented in BioGeoBEARS.** Highest ranked model indicated in red.

| **Model** | **LnL** | **#params** | ***d*** | ***e*** | ***j*** | **AIC** | **AIC_wt** | **AIC** | **AICc** | **ΔAICc** | **rel.LnL** | **AICc_wt** | **%rel.wt** |
| --- | --- | --- | --- | --- | --- | --- | --- | --- | --- | --- | --- | --- | --- |
| DEC | -16.60 | 2 | 2.40E-02 | 1.00E-12 | 0 | 37.20 | 1.7E-03 | 37.20 | 37.771429 | 14.459894 | 7.425592E-04 | 5.266836E-04 | 0.05 |
| DEC+J | -10.15 | 3 | 1.00E-12 | 1.00E-12 | 0.075 | 26.30 | 3.9E-01 | 26.30 | 27.157143 | 3.845609 | 1.461964E-01 | 1.062705E-01 | 10.63 |
| DIVALIKE | -18.02 | 2 | 4.00E-02 | 1.00E-12 | 0 | 40.04 | 4.0E-04 | 40.04 | 40.611429 | 17.299894 | 1.751361E-04 | 1.273068E-04 | 0.01 |
| DIVALIKE+J | -10.22 | 3 | 1.00E-12 | 1.00E-12 | 0.082 | 26.45 | 3.6E-01 | 26.44 | 27.297143 | 3.985609 | 1.363126E-01 | 9.908594E-02 | 9.91 |
| BAYAREALIKE | -23.29 | 2 | 3.60E-02 | 6.10E-02 | 0 | 50.58 | 2.1E-06 | 50.58 | 51.151429 | 27.839894 | 9.008319E-07 | 6.548166E-07 | 6.55E-05 |
| BAYAREALIKE+J | -10.61 | 3 | 1.00E-07 | 1.00E-07 | 0.079 | 27.22 | 2.5E-01 | 27.22 | 28.077143 | 4.765609 | 9.229140E-02 | 6.708682E-02 | 6.72 |
| **MK** | **-10.51** | **1** | **1.00E-12** | **1.00E-12** | **0** |  |  | **23.03** | **23.311534** | **0** | **1** | **7.269021E-01** | **72.69** |

LnL = log-likelihood, #params = number of parameters, *d* = dispersal parameter, *e* = extinction parameter, *j* = jump dispersal parameter, AIC/c = Akaike’s Information Criterion/corrected for sample size, wt = weight, rel. = relative.

**Table S5.** **Summary of mtDNA population genetics and expansion test statistics (Tajima’s *D*, Fu’s *Fs*) for major lineages in the *Gehyra australis* group.** Taxa and lineages shaded in grey are saxicoline, unshaded are generalists. Highlighted in red are lineages that show a signature of expansion (significant *P*-values).

| **Clade** | ***n*** | ***n* (lineages)** | ***π*** | ***Dxy*** | **Tajima's *D*** | ***P_D_*** | **Fu's *Fs*** | ***P_Fs_*** | ***R*2** | ***P*_R2_** |
| --- | --- | --- | --- | --- | --- | --- | --- | --- | --- | --- |
| *G. australis* | 255 | 4 | 0.074 | 0.161 | 0.263 | 0.672 | **-28.635** | **0.001** | 0.112 | 0.915 |
| *G. borroloola* | 38 | 2 | 0.043 | 0.130 | 0.643 | 0.782 | 5.983 | 0.957 | 0.143 | 0.833 |
| *G. catenata* | 3 | 1 | 0.049 | 0.160 | NA | NA | NA | NA | NA | NA |
| CYsp | 29 | 1 | 0.092 | 0.242 | -0.082 | 0.497 | 5.100 | 0.963 | 0.137 | 0.731 |
| *G. dubia* | 59 | 2 | 0.085 | 0.160 | 0.153 | 0.639 | -3.218 | 0.186 | 0.136 | 0.887 |
| Groote | 4 | 1 | 0.004 | 0.151 | NA | NA | NA | NA | NA | NA |
| *G. koira* | 254 | 5 | 0.088 | 0.151 | 0.219 | 0.678 | -7.278 | 0.134 | 0.110 | 0.891 |
| *G. pamela* | 35 | 5 | 0.068 | 0.208 | -0.657 | 0.292 | 3.538 | 0.905 | 0.110 | 0.514 |
| *G. robusta* | 86 | 5 | 0.079 | 0.130 | -0.033 | 0.546 | 3.120 | 0.832 | 0.117 | 0.761 |
| **Total** | 763 |  |  |  |  |  |  |  |  |  |
| **Lineage** |  |  |  |  |  |  |  |  |  |  |
| aus1 | 127 |  | 0.023 | 0.094 | -1.043 | 0.162 | **-32.944** | **0.000** | 0.067 | 0.261 |
| aus2 | 73 |  | 0.029 | 0.094 | 0.578 | 0.775 | 1.169 | 0.691 | 0.361 | 0.956 |
| aus3 | 41 |  | 0.032 | 0.108 | 0.325 | 0.675 | -0.748 | 0.398 | 0.130 | 0.733 |
| aus4 | 14 |  | 0.010 | 0.109 | -0.135 | 0.470 | -0.026 | 0.492 | 0.139 | 0.307 |
| bor1 | 32 |  | 0.025 | 0.090 | 0.496 | 0.736 | 4.473 | 0.932 | 0.137 | 0.750 |
| bor2 | 6 |  | 0.001 | 0.090 | NA | NA | NA | NA | NA | NA |
| *G. catenata* | 3 |  | 0.049 | 0.161 | NA | NA | NA | NA | NA | NA |
| CYsp | 29 |  | 0.092 | 0.239 | -0.082 | 0.497 | 5.100 | 0.963 | 0.137 | 0.731 |
| dub1 | 33 |  | 0.045 | 0.123 | -0.623 | 0.278 | -4.324 | 0.088 | 0.108 | 0.445 |
| dub2 | 26 |  | 0.050 | 0.123 | 0.006 | 0.557 | 1.369 | 0.744 | 0.134 | 0.692 |
| Groote | 4 |  | 0.004 | 0.116 | NA | NA | NA | NA | NA | NA |
| *G. ipsa* | 4 |  | 0.002 | 0.060 | NA | NA | NA | NA | NA | NA |
| koira1 | 176 |  | 0.060 | 0.064 | -0.015 | 0.575 | -4.803 | 0.187 | 0.102 | 0.764 |
| koira2 | 15 |  | 0.007 | 0.085 | -0.216 | 0.485 | -1.172 | 0.234 | 0.124 | 0.240 |
| koira3 | 18 |  | 0.030 | 0.064 | 0.694 | 0.800 | 4.728 | 0.968 | 0.166 | 0.805 |
| koira4 | 41 |  | 0.027 | 0.060 | 0.670 | 0.782 | 2.628 | 0.846 | 0.134 | 0.773 |
| pam1 | 25 |  | 0.028 | 0.112 | 0.080 | 0.592 | 1.879 | 0.781 | 0.130 | 0.623 |
| pam2 | 6 |  | 0.001 | 0.112 | NA | NA | NA | NA | NA | NA |
| pam3 | 1 |  | NA | 0.123 | NA | NA | NA | NA | NA | NA |
| pam4 | 1 |  | NA | 0.135 | NA | NA | NA | NA | NA | NA |
| pam5 | 2 |  | 0.031 | 0.113 | NA | NA | NA | NA | NA | NA |
| rob1 | 23 |  | 0.034 | 0.093 | 0.569 | 0.755 | 3.071 | 0.909 | 0.153 | 0.788 |
| rob2 | 10 |  | 0.019 | 0.093 | 0.128 | 0.593 | 3.228 | 0.924 | 0.172 | 0.607 |
| rob3 | 8 |  | 0.017 | 0.086 | NA | NA | NA | NA | NA | NA |
| rob4 | 3 |  | 0.000 | 0.086 | NA | NA | NA | NA | NA | NA |
| rob5 | 42 |  | 0.015 | 0.116 | -0.951 | 0.174 | -1.317 | 0.349 | 0.084 | 0.223 |

*n*, number of individuals; *π*, average Tamura-Nei corrected pairwise divergence between individuals; *Dxy*, average pairwise divergence to nearest relative; *P_D_, P_Fs_, P_R2_*, significance test *P*-values for each statistic written in sub-script; *R2*, Ramos-Onsins and Rozas R2 value; NA, value not calculable from < 10 individuals.

**Table S6.** **Comparison of mean Diversification Rate (DR) statistic estimates for the *australis* group StarBEAST2 phylogeny, stratified by ecology (habitat).** My = million years.

| **Grouping** | DR statistic (lineages/My) mean with 95% CI:  **[all *australis* group]** | DR statistic (lineages/My) mean with 95% CI:  **[no CYsp/*catenata*/*dubia*]** |
| --- | --- | --- |
| Whole group | 0.365 (0.162–0.626) | 0.417 (0.237–0.645) |
| Saxicoline | 0.391 (0.209–0.596) | 0.405 (0.234–0.593) |
| Generalist | 0.322 (0.126–0.567) | 0.450 (0.309–0.610) |

**Table S7.** **Loadings, variance and percentage of data variance explained from the principal components analyses (PCAs).** Analyses conducted on all log-transformed morphological traits (“sPCA”), and on the residuals of body shape traits against SVL (“rPCA”). Values in red highlight the traits with the strongest loadings on each principal component axis.

| **Trait** | **sPC1** | **sPC2** | **sPC3** | **rPC1** | **rPC2** | **rPC3** |
| --- | --- | --- | --- | --- | --- | --- |
| Snout-vent length (SVL) | -0.30 | 0.00 | 0.00 | NA | NA | NA |
| Trunk length | -0.29 | -0.17 | 0.25 | 0.39 | 0.11 | -0.17 |
| Trunk width | -0.29 | -0.12 | **-0.45** | -0.25 | **-0.43** | 0.00 |
| Foreleg length | -0.30 | 0.00 | -0.12 | -0.22 | 0.32 | **-0.46** |
| Hindleg length | -0.30 | 0.00 | 0.00 | 0.00 | **0.50** | -0.35 |
| Head length | -0.30 | 0.00 | -0.27 | **-0.45** | 0.00 | -0.12 |
| Head depth | -0.29 | -0.19 | 0.17 | 0.22 | -0.23 | **-0.44** |
| Head width | -0.30 | 0.00 | -0.27 | -0.38 | -0.28 | -0.27 |
| Snout length | -0.29 | 0.18 | -0.17 | -0.31 | 0.36 | -0.15 |
| Snout depth | -0.30 | -0.25 | 0.00 | 0.20 | -0.27 | **-0.53** |
| Toe length | -0.28 | -0.13 | **0.69** | **0.42** | 0.17 | -0.15 |
| Lamellae | -0.22 | **0.90** | 0.19 | -0.15 | 0.30 | 0.18 |
| Component variance | 3.28 | 0.75 | 0.58 | 1.98 | 1.55 | 1.43 |
| % data variance explained | 90 | 5 | 3 | 35 | 22 | 19 |

**Table S8.** **Multivariate tests for morphological differentiation between habitat types in the *Gehyra australis* group.** Analysed using multivariate morphometric data analyses and ANOVA (phylogenetic or non-phylogenetic, based on tests for phylogenetic signal). Significant *P*-value highlighted in red.

| **Variable** | **Test (saxicoline vs. generalist)** | ***P*-value** |
| --- | --- | --- |
| sPC1 (SVL) | ANOVA (non-phylogenetic) | 0.092 |
| sPC2 (Lamellae) | ANOVA (phylogenetic) | 0.104 |
| sPC3 (ToeL) | ANOVA (phylogenetic) | 0.144 |
| rPC1 (HeadL, ToeL) | ANOVA (phylogenetic) | **0.015** |
| rPC2 (HindlegL, TrunkW) | ANOVA (non-phylogenetic) | 0.093 |
| rPC3 (SnoutD, HeadD, ForelegL) | ANOVA (non-phylogenetic) | 0.658 |

**Table S9.** **Univariate tests for morphological differentiation between habitat types in the *Gehyra australis* group.** Analysed using univariate morphological trait data and phylogenetic least squares (PGLS) regression and the most likely model of trait evolution (BM = Brownian Motion, OU = Ornstein-Uhelenbeck). Significant *P*-values highlighted in red.

| **Variable** | **Test (saxicoline vs. generalist)** | ***P*-value** |
| --- | --- | --- |
| SVL | PGLS (OU) | **0.028** |
| TrunkL | PGLS (BM) | 0.687 |
| TrunkW | PGLS (OU) | 0.586 |
| ForelegL | PGLS (OU) | **0.039** |
| HindlegL | PGLS (OU) | 0.053 |
| HeadL | PGLS (BM) | **0.040** |
| HeadD | PGLS (OU) | **0.031** |
| HeadW | PGLS (BM) | 0.297 |
| SnoutL | PGLS (OU) | **0.008** |
| SnoutD | PGLS (BM) | **0.010** |
| ToeL | PGLS (BM) | **0.008** |
| Lamellae | PGLS (BM) | 0.094 |
