# Supplementary material for: On and off the rocks: persistence and ecological diversification in a tropical Australian lizard radiation
Source: BMC Evol Biol. 2019 Mar 20;19:81. doi: 10.1186/s12862-019-1408-1 (PMC6427882; doi:10.1186/s12862-019-1408-1)
Supplement: Supplementary file 5 — Figure S1. Geographic distribution and lineage allocation for samples from the Gehyra australis group included in exon capture. Figure S2. Topology and support values estimated by ASTRAL-II from 556 IQ-TREE gene trees for the Gehyra australis group. Figure S3. ASTRAL-III quartet support mapped onto species tree for the Gehyra australis group. Figure S4. Mean and ranges of values for morphological characters that showed evidence of significant variation across saxicoline and generalist taxa (according to PGLS) in the Gehyra australis group. Figure S5. Morphological regime shifts within the Gehyra australis group inferred by SURFACE based on an analysis including SVL (log-transformed) and 11 other size-corrected morphological characters. Figure S6. Morphological regime shifts within the Gehyra australis group inferred by l1OU based on an analysis including SVL (log-transformed) and 11 other size-corrected morphological characters. (DOC 999 kb) [file 12862_2019_1408_MOESM5_ESM.doc]

*BMC Evolutionary Biology*

**ADDITIONAL FILE 5**

**On and off the rocks: persistence and ecological diversification in a tropical Australian lizard radiation.**

Paul M. Oliver, Lauren G. Ashman, Sarah Bank, Rebecca J. Laver, Renae C. Pratt, Leonardo G. Tedeschi and Craig C. Moritz

**Additional file 5:** geographic distribution of samples included in exon capture (Fig. S1); ASTRAL-II multi-species coalescent tree (Fig. S2); ASTRAL-III quartet support on the phylogenetic species tree (Fig. S3); comparison of morphological characters between habitat types (saxicoline vs. generalists) (Fig. S4); morphological regime shifts across the phylogeny inferred by SURFACE (Fig. S5); morphological regime shifts across the phylogeny inferred by l1OU (Fig. S6).

**Figure S1.** Geographic distribution and lineage allocation for samples from the *Gehyra australis* group included in exon capture.

**Figure S2.** Topology and support values (multilocus bootstrapping) estimated by ASTRAL-II from 556 IQ-TREE gene trees for the *Gehyra australis* group.

**Figure S3.** ASTRAL-III Quartet supports (out of 100) mapped onto the species tree for the *Gehyra australis* group. Scale bar indicates substitutions per site.


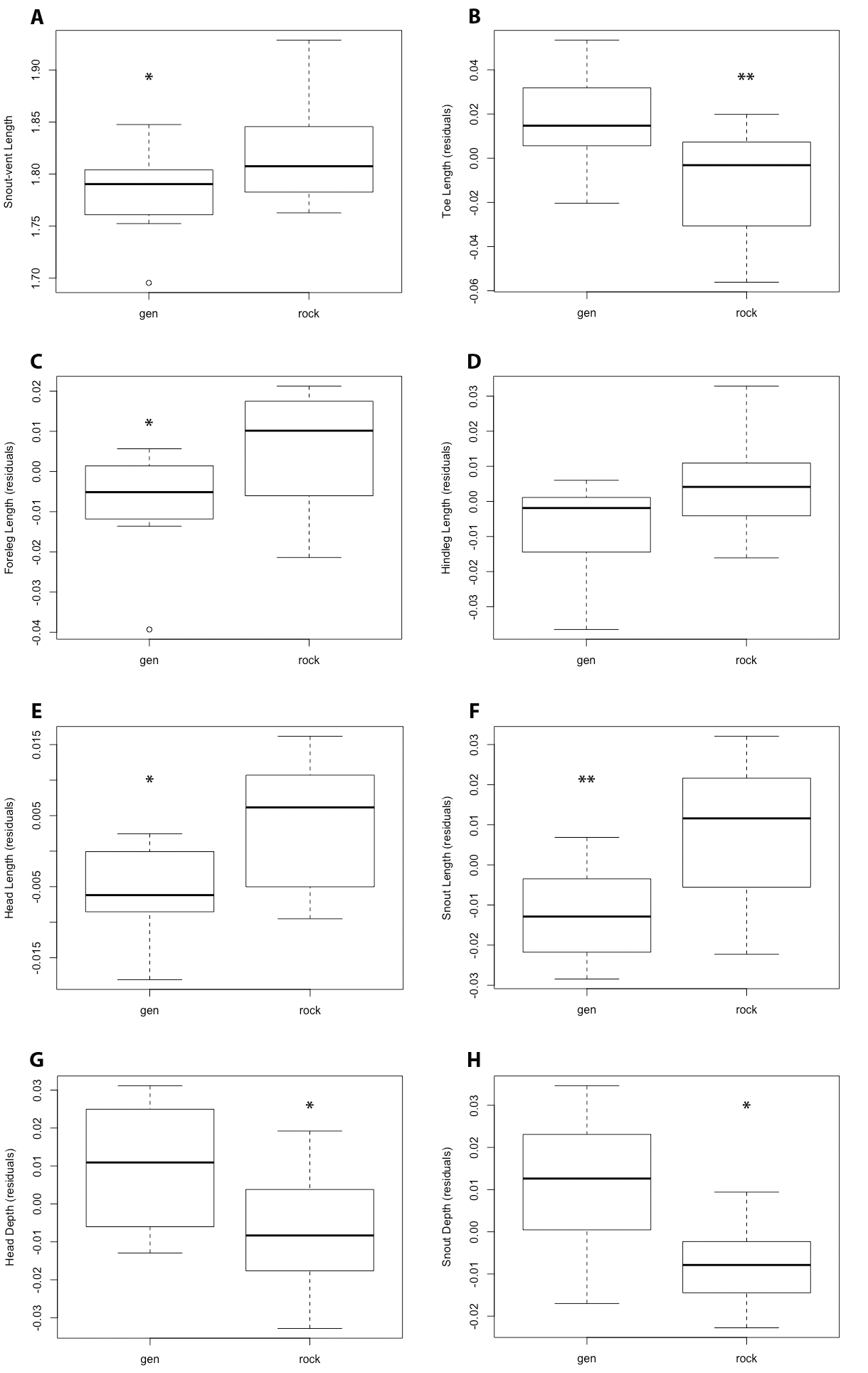


**Figure S4.** Morphological character means and ranges for saxicoline (rock) vs. generalist (gen) taxa of the *Gehyra australis* group, analysed for traits which showed evidence of significant variation between habitat types (according to Phylogenetic Generalized Least Squares [PGLS] regression analyses). * = *P*-value < 0.05, ** = *P*-value < 0.01.

**Figure S5.** Morphological regime shifts within the *Gehyra australis* group inferred by SURFACE, based on an analysis including SVL (log-transformed) and 11 other size-corrected morphological characters. Numbered circles indicate points of regime shifts across the phylogeny.

**Figure S6.** Morphological regime shifts within the *Gehyra australis* group inferred by lIOU based on an analysis including SVL (log-transformed) and 11 other size-corrected morphological characters. Regime shifts denoted on tree by * with associated support values below.
